# Immunization costs, from evidence to policy: Findings from a nationally representative costing study and policy translation effort in Tanzania

**DOI:** 10.1016/j.vaccine.2020.10.004

**Published:** 2020-11-10

**Authors:** Kelsey Vaughan, Emma Clarke-Deelder, Kassimu Tani, Dafrossa Lyimo, Alex Mphuru, Fatuma Manzi, Carl Schütte, Annette Ozaltin

**Affiliations:** a ThinkWell, Washington, DC, USA; b Harvard T.H. Chan School of Public Health, Department of Global Health and Population, Boston, MA, USA; c Ifakara Health Institute, Dar es Salaam, Tanzania; d Immunization and Vaccines Development (IVD), Ministry of Health, Community Development, Gender, Elderly and Children, Dar es Salaam, Tanzania; e Genesis Analytics, Johannesburg, South Africa

**Keywords:** Expanded programme on immunization, Immunization, Vaccination, Immunization delivery costs, Immunization economics, Costing

## Abstract

**Introduction:**

Information on the costs of routine immunization programs is needed for budgeting, planning, and domestic resource mobilization. This information is particularly important for countries such as Tanzania that are preparing to transition out of support from Gavi, the Vaccine Alliance. This study aimed to estimate the total and unit costs for of child immunization in Tanzania from July 2016 to June 2017 and make this evidence available to key stakeholders.

**Methods:**

We used an ingredients-based approach to collect routine immunization cost data from the facility, district, regional, and national levels. We collected data on the cost of vaccines as well as non-vaccine delivery costs. We estimated total and unit costs from a provider perspective for each level and overall, and examined how costs varied by delivery strategy, geographic area, and facility-level service delivery volume. An evidence-to-policy plan identified key opportunities and stakeholders to target to facilitate the use of results.

**Results:**

The total annual economic cost of the immunization program, inclusive of vaccines, was estimated to be US$138 million (95% CI: 133, 144), or $4.32 ($3.72, $4.98) per dose. The delivery costs made up $45 million (38, 52), or $1.38 (1.06, 1.70) per dose. The costs of facility-based delivery were similar in urban and rural areas, but the costs of outreach delivery were higher in rural areas than in urban areas. The facility-level delivery cost per dose decreased with the facility service delivery volume.

**Discussion:**

We estimated the costs of the routine immunization program in Tanzania, where no immunization costing study had been conducted for five years. These estimates can inform the program’s budgeting and planning as Tanzania prepares to transition out of Gavi support. Next steps for evidence-to-policy translation have been identified, including technical support requirements for policy advocacy and planning.

## Highlights

• Delivery costs represent 33% of total immunization program costs in Tanzania.
• Costs are higher for outreach than for facility-based delivery.
• We used calibration methods to estimate unit and total costs.
• This work will inform domestic resource advocacy and planning.

## 1 Introduction

Over the past ten years, routine immunization coverage in the United Republic of Tanzania has increased considerably, from 86% to 98% coverage of the third dose of the diphtheria-tetanus-pertussis (DTP) vaccine, and from 88% to 99% coverage of the first dose of the measles-rubella vaccine [1]. To maintain these gains and continue scaling up newer vaccines, Tanzania’s immunization program will need sufficient and reliable funding. This is particularly important as Tanzania prepares to transition out of support from Gavi, the Vaccine Alliance, towards increased domestic financing of immunization. Vaccine costs and their associated delivery costs are likely to increase for countries like Tanzania during and after their transition from Gavi support [2], and additional funding will likely be needed to support the introduction of new vaccines. Cost data are required to support domestic resource mobilization for the delivery of the existing schedule of vaccines, and to inform new vaccine introduction planning [3], [4], [5], [6]. In addition, understanding how costs vary across health facilities can inform efforts to improve the efficiency of delivery [7], [8].

The currently available cost data is insufficient to support budgeting and planning for Tanzania’s immunization program. Previous immunization costing studies in Tanzania focused only on specific antigens or limited geographic areas, and the most recent study was five years ago [9], [10], [11], [12], [13], [14]. In a national planning exercise conducted to develop a comprehensive Multi-Year Plan (cMYP) for immunization from 2016 to 2020, the Government of Tanzania identified the generation of new cost evidence as a priority. In addition to generating new evidence, there is a need to translate evidence so that it can be used effectively in decision-making processes and for program management. This was identified as a key gap in previous global immunization costing efforts, where robust research was not often taken up by country governments to support planning, budgeting and decision-making processes [15], [16].

The aim of the study was to estimate the total and unit costs of routine childhood vaccination in Tanzania, with and without vaccine costs, for the period from July 2016 to June 2017, and to translate these estimates for use in policymaking, budgeting, and other decision-making efforts.

## 2 Methods

### 2.1 Study setting

This study was implemented in the United Republic of Tanzania, comprised of mainland Tanzania (population 52.6 million) and the island of Zanzibar (1.6 million, 2018) [17]. Nationwide coverage of DTP3 in Tanzania is 98%, while coverage of Measles-Rubella 2nd dose (MR2) is 84% [1]. The predominant vaccine delivery strategy is fixed facility-based delivery, but non-facility-based delivery strategies also play an important role important as 71% of the population lives in rural and hard-to-reach areas and 17% of the population is nomadic (The United Republic of Tanzania, 2013). The Government of Tanzania uses outreach and mobile delivery strategies to reach populations that cannot be reached through facility-based delivery. Outreach is used to reach populations living more than 5 but less than 10 km from health facilities; transport is usually by motorcycle, bus or taxi and without an overnight stay. Mobile clinics are used to reach populations living more than 10 km from a health facility, such as nomadic and hard-to-reach communities; transport is usually with a vehicle and health workers typically stay overnight.

Tanzania’s health system, including the Immunization and Vaccine Development Program (IVD), was decentralized in the 1990s [18]. The national level retained responsibility for policymaking, developing strategies, issuing guidelines, procuring vaccines and injection supplies and transporting them to regional level, storing and managing stock nationally, and collating and reporting on coverage and other performance data [19]. Human resources are also centrally managed by the President’s Office, Public Service Management and Good Governance, and financed by the Ministry of Finance. Regions are responsible for managing stock at regional stores, the supply chain to districts and supervision, while districts are responsible for the supply chain to facilities, supervision, ongoing adverse event and disease surveillance and service delivery.

At the time of our study, the IVD provided six vaccines to children under 18 months of age (Table 1). The IVD has since also introduced the inactivated polio vaccine into the routine schedule. Currently, Tanzania receives support from Gavi, the Vaccine Alliance, to cover a portion of the costs of the pentavalent, pneumococcal, rotavirus, measles-rubella, and inactivated polio vaccines. The government is largely responsible for the delivery or operational costs. Delivery costs are included in the budget of the Comprehensive Council Health Plan (CCHP) prepared by local governments. However, uncertainty regarding operational costs and a target-driven need for operational resources has typically led to budgeting based on historical expenditures plus an inflationary increase [20].

**Table 1:** Overview of Tanzania’s immunization schedule, up to 18 months (2016).

Antigens	Target Age
• Oral polio vaccine – dose 0 (OPV0)	At birth up to 14 days
• Bacillius-Calmette-Guérin (BCG)	At birth or first contact
• Oral polio vaccine – dose 1 (OPV1) • Diptheria-Tetanus-Pertussis-Hepatitis B-Haemophilus influenzae type b – dose 1 (DTP-HepB-Hib1) • 13-valent pneumococcal – dose 1 (PCV13-1) • Rotavirus – dose 1 (Rota1)	6 weeks
• Oral polio vaccine – dose 2 (OPV2) • Diptheria-Tetanus-Pertussis-Hepatitis B-Haemophilus influenzae type b – dose 2 (DTP-HepB-Hib2) • 13-valent pneumococcal – dose 2 (PCV13-2) • Rotavirus – dose 2 (Rota2)	10 weeks
• Oral polio vaccine – dose 3 (OPV3) • Diptheria-Tetanus-Pertussis-Hepatitis B-Haemophilus influenzae type b – dose 3 (DTP-HepB-Hib3) • 13-valent pneumococcal – dose 3 (PCV13-3)	14 weeks
• Measles-Rubella – dose 1 (MR1)	9 months
• Measles-Rubella – dose 2 (MR2)	18 months

### 2.2 Scope of costing exercise

The goal of this study was to estimate the total and unit costs of routine immunization in Tanzania for a 1-year period, disaggregated into vaccine and (non-vaccine) delivery costs. We estimated two types of unit costs: the cost per dose, and the cost per fully immunized child (FIC). We defined the cost per FIC as the cost per child that received a second dose of the measles-rubella vaccine (MR2). While in some contexts a FIC is defined as a child who has received a third dose of the diptheria-tetanus-pertussis (DTP) vaccine, we used the definition preferred by the Ministry of Health in Tanzania to maximize the utility of our findings for policymakers. We included routine immunization services delivered to children up to 18 months in rural and urban areas at current coverage levels. We stratified results by delivery strategies (fixed facility delivery, outreach and mobile), geographic area type (urban, rural with nomadic populations, and rural without nomadic populations), and district. We also examined the association between facility-level delivery volume and facility-level unit cost.

Methods were adapted from those used in the Expanded Program on Immunization Costing and Financing (EPIC) studies [21], [22]. We estimated economic costs incurred during the financial year July 2016 to June 2017 from a provider perspective. We defined economic costs as financial outlays incurred by the government plus opportunity costs of health worker time and any donated items, with capital items depreciated using a discount rate. Costs from all levels of the health system, including facility, district, region and national levels, were included. We used ingredients-based costing, where the full costs and quantities of inputs (cost line items, e.g., human resources, cold chain energy costs, buildings) used for routine immunization activities (e.g., outreach service delivery, social mobilization, supervision) were valued (Box 1).

Box 1 **Cost line items and activities.**

Cost line items: (1) labor, (2) per diem and travel allowances, (3) vaccines, (4) vaccine injection and safety supplies, (5) other supplies, (6) transport and fuel, (7) vehicle maintenance, (8) cold chain energy, (9) printing, (10) utilities and communication, (11) other recurrent costs, (12) cold chain equipment, (13) vehicles, (14) lab equipment, (15) other equipment, (16) buildings, (17) other capital items.

Cost activities: (1) routine facility-based service delivery, (2) record keeping, HMIS, monitoring and evaluation, (3) supervision, (4) outreach service delivery, (5) mobile service delivery, (6) training, (7) social mobilization and advocacy, (8) surveillance, (9) vaccine collection, distribution and storage, (10) program management, (11) cold chain maintenance, (12) other.

Shared inputs including labor time, equipment and vehicles were allocated to routine immunization and specific immunization activities on the basis of responses from staff on usage. Capital costs such as equipment, training and vehicles were annualized based on useful lifetimes, adapted from recently validated estimates for immunization, other costing studies and WHO CHOICE [21], [23], [24].

### 2.3 Sample

We developed a nationally representative sample of zones, regions, districts, and facilities. Tanzania has seven geographic zones which are divided into 30 regions and 169 districts. We first categorized the seven zones into four strata defined by DTP3 coverage levels as reported in the Demographic and Health Survey (DHS) 2015–16: high (more than 80%), high-medium (76–80%), medium (70–75%) and low (less than 70%) [25]. From each coverage stratum we selected one region. Within each selected region, we randomly selected one urban district and one rural district without nomadic people in the catchment population. We also purposively selected one rural district that included nomadic people in the catchment population. Within each selected district we randomly selected four health facilities (or five, in districts with nomadic populations). Since we expected costs not to be normally distributed but right-skewed, oversampling districts with nomadic populations would produce a sample that would include more observations of facilities associated with the right tail [26]). The resulting sample included four regions, 12 districts and 54 facilities. The four selected regions were Mbeya (in the South West Highlands of Tanzania, with a population of 2.7 M in the 2012 census, approximately 67% of people living in rural areas, approximately 13% living with severe poverty, and 67% of children receiving a full course of basic vaccines in 2015–2016), Morogoro (in the Eastern zone of Tanzania, with a population of 2.1 M in the 2012 census, approximately 71% of people living in rural areas, approximately 9% of people living in severe poverty, and 81% of children receiving a full course of basic vaccines), Mtwara (in the Southern zone on Tanzania’s border with Mozambique, with a population of 1.3 M, approximately 71% of people living in rural areas, 14% of people living with severe poverty, and 79% of children receiving a full course of basic vaccines), and Simiyu (in the Lake zone in the north of Tanzania, with a population of 1.6 M, approximately 93% of people living in rural areas, 22.6% of people living with severe poverty and 68% of children receiving a full course of basic vaccines) [25], [27], [28], [29]. Since three facilities were excluded before analysis due to large amounts of missing data, the analytic sample ultimately included four regions, 12 districts, and 51 facilities.

### 2.4 Data collection

Standard data collection instruments were adapted from EPIC [21]. These were tailored to the study and local context based on the study protocol, further contextualized during a pre-pilot interview with facility staff, and revised after pilot testing. For example, adaptations included revising the list of vaccines to match those delivered in Tanzania during the study period and revising the roles of different cadres of health workers to align with the way roles are defined in the Tanzanian health system. Data on costs from July 2016 through June 2017 were collected retrospectively over the period February to June 2018 through interviews with staff, as well as extraction from existing reports, program records, and the health information system. Additional details on data sources and unit prices are included in the Supplementary Appendix (Table S1, Table S2, and Table S3).

### 2.5 Data cleaning and analysis

Immediately following data collection, data were entered in a Microsoft Excel-database tool previously used for the EPIC studies, which was modified to mimic our questionnaires. We performed validation checks to ensure data were entered properly and that answers were within a plausible range. We also investigated any findings that appeared on visual inspection to be problematic to identify possible data entry errors. We used imputation techniques to fill several data gaps in the number of doses delivered and the breakdown between delivery strategies, using data from available months or similar facilities.

We began our analysis by describing the characteristics of the sampled sites. We then estimated total and unit economic costs (per dose and per FIC) in local currency. At the facility, district, and regional levels, total and unit costs were estimated using calibration weights, which adjust inverse probability of sampling weights (IPWs) to account for differences between sample and population characteristics [30]. This method has been shown to reduce bias and variance in cost estimates for healthcare service delivery programs [31], [32]. We implemented calibration weighting using the survey package in R [33]. Total delivery volumes of DTP3 and MR2 were used as calibration targets.

At the national level, the calibration approach was not relevant as we were not analyzing data from a sample. To estimate the national-level unit costs, we divided the national-level total costs by an estimate of total doses delivered generated using the facility-level data. We estimated the national-level total doses delivered from the facility-level data because we did not have complete primary data on the total vaccine delivery volume at the national level.

We calculated 95% confidence intervals for all estimates, reflecting the use of calibration weighting and the clustered sampling design.

We converted all estimates to 2016 US$ using a conversion rate of 1 US$ = 2177 Tanzania shillings (World Bank official exchange rate, period average for 2016). We report estimates of economic costs.

We examined the association between service delivery volume and unit costs at the facility level using linear regression models, regressing the logged unit cost on the logged service delivery volume. We calculated the percent change in unit costs associated with a 10% increase in service delivery volume by exponentiating regression coefficients and multiplying by 10.

### 2.6 Policy translation

At the start of this study in March 2017, the research team met with a group of local stakeholders to discuss data needs, following up on the prioritization of cost data in Tanzania’s 2016–2020 cMYP. We then developed a detailed evidence-to-policy plan for use of the results from this study. The plan presented a condensed version of key research findings, consolidated feedback from local stakeholders about the evidence-to-policy trajectory in Tanzania, noted potential use cases for the costing findings including critical points at which the evidence is needed (in meetings, but typically earlier in estimation and modeling exercises) and the key stakeholders for each use case, and outlined needed action for policy translation of the cost evidence, such as further technical support. After data collection and analysis were completed, a dissemination event was held in Tanzania in November 2019. Participants were asked for feedback on the optimal next steps to ensure successful evidence-to-policy translation. We summarized key themes in participants’ responses.

### 2.7 Ethical approval

Ethical approval was granted on 19 February 2018 by the National Institute for Medical Research (NIMR) in Tanzania with reference number NIMR/HQ/R.8a/Vol.IX/2702.

## 3 Findings

### 3.1 Description of the sample

Our analytical sample included 51 facilities, 12 districts, 4 regions, and national-level data. While the original sample included 54 facilities, three sampled facilities were dropped during analysis due to large amounts of missing data. A description of the analytic sample (i.e. the 51 sites remaining in the sample after three were dropped), showing sample characteristics before the calibration technique was applied, is included in Table 2. Delivery volumes in the selected facilities ranged from 566 to 52,600 doses per year for facility-based delivery, and 40–3300 doses per year for outreach-based delivery. The average annual delivery volume, including both facility-based and outreach delivery, was 5704 doses in the facilities in rural areas without nomadic populations; 7050 doses in the facilities in rural areas with nomadic populations; and 11,763 doses in facilities in urban areas. Three urban facilities in the sample had particularly high delivery volumes (over 20,000 doses per year). Across the sampled facilities, the facility-level delivery costs per dose ranged from $0.83 to $7.11 for facility-based delivery, and $2.57–$26.75 for outreach. District-level costs per dose ranged from $0.11 to $0.48, while regional-level costs per dose ranged from $0.02 to $0.03. At the national level, a reported 21 million doses were delivered, but this excluded doses of OPV0, OPV1, OPV2, and DTP2 because the WHO-UNICEF Joint Reporting Form, which was the source for these data, does not include these vaccines as indicators to report. Therefore, for unit cost analyses for the national level, we estimated the total number of doses delivered (inclusive of OPV0, OPV1, OPV2, and DTP2) based on the data reported at the facility level.

**Table 2:** Description of volumes and economic unit costs per dose in the sampled sites (2016 US$).

		Facility-level						District level	Regional level
		Total	By delivery type		By geography type				
			Facility	Outreach^1^	Urban	Rural (without nomadic population)	Rural (with nomadic populations)		
	N (sites)	51	51	27	17	20	14	12	4
Doses delivered^2^	Minimum	566	566	43	566	809	2,234	67,002	723,402
	Maximum	53,235	52,603	3,300	53,235	19,488	19,566	259,786	1,146,887
	Median	5,830	4,691	428	7,883	4,093	5,180	186,258	956,656
	Mean^3^	8,093	7,663	811	11,763	5,703	7,049	180,654	945,900
	Standard deviation	8,738	8,718	970	12,875	4,911	5,178	54,169	173,362
Economic cost per dose (2016 US$)^4^	Minimum	0.83	0.83	2.57	0.83	2.01	1.54	0.11	0.02
	Maximum	7.11	7.11	26.75	7.11	6.79	5.43	0.48	0.03
	Median	3.72	3.50	5.65	3.50	4.01	3.66	0.20	0.03
	Mean^3^	3.47	3.41	4.63	3.39	3.69	3.69	0.19	0.03
	Standard deviation	1.51	1.47	6.27	1.87	1.32	1.16	0.10	<0.01

Notes:

1 This only includes data from the 27 sites reporting having done outreach.

2 This includes all of the vaccine doses delivered to children under the age of 18 months, including: oral polio vaccine, Bacillus-Calmette-Guérin vaccine, Diptheria-Tetanus-Pertussis-Hepatitis B-Haemophilus influenzae type b vaccine, pneumococcal vaccine, rotavirus vaccine, and measles-rubella vaccine.

3 This mean describes the sample only. The mean economic cost per dose presented in this table is calculated by dividing the total cost by the total delivery volume among the sampled sites. This mean should not be reported as the estimate for the full country because it does not reflect appropriate sampling weighting.

4 The numerator is the total cost of delivering vaccines to children under 18 months, and the denominator is the total number of vaccine doses delivered to children under 18 months.

### 3.2 Total costs

The estimated total delivery cost of the routine immunization program (exclusive of vaccines, injection supplies and paid human resources (labor) costs) was $44.6 million (95% CI: 37.6–51.5) including facility, district, regional and national level costs (Table 3). This delivery cost represented 32% of the estimated total program cost of $138.2 million (95% CI: 132.6–143.8), while vaccines and injection supplies represented 68% of the total. By level of the system, the total cost was comprised of facility-level costs (87%), district-level costs (4%), regional costs (1%), and national costs (8%). The delivery costs (exclusive of vaccines and injection supplies) were comprised of facility-level costs (83%), district-level costs (14%), regional costs (2%) and national-level costs (1%).

**Table 3:** Estimated facility, district, regional and national-level economic cost per dose (2016 US$).

Level	Vaccines and injection supplies^1^ (95% CI)^2^	Delivery costs (95% CI)	Total (95% CI)
Facility	2.61 (2.26–2.96)	1.15 (0.83–1.47)	3.76 (3.16–4.37)
District	—	0.19 (0.17–0.21)	0.19 (0.17–0.21)
Regional	—	0.03 (0.03–0.04)	0.03 (0.03–0.04)
National	0.33	<0.01	0.34
Total	2.94 (2.59–3.29)	1.38 (1.06–1.70)	4.32 (3.72–4.93)

Notes:

Table shows average economic costs per vaccine dose delivered. The denominator includes all of the vaccine doses delivered to children under the age of 18 months, including: oral polio vaccine, Bacillus-Calmette-Guérin vaccine, Diptheria-Tetanus-Pertussis-Hepatitis B-Haemophilus influenzae type b vaccine, pneumococcal vaccine, rotavirus vaccine, and measles-rubella vaccine. Costs are disaggregated into (1) vaccine and injection supplies, and (2) delivery costs.

1 Vaccine costs are reported only at facility level as this is where vaccine delivery occurs, but in reality these costs were incurred at national level.

2 95% Confidence Interval.

### 3.3 Costs per facility

The average delivery costs per facility per year were highest at facilities in urban areas ($6891 per year, 95% CI: 5466–8317), compared with facilities in rural areas without nomadic populations ($4569 per year, 95% CI: 3289–5489) and facilities in rural areas with nomadic populations ($4982 per year, 95% CI: 3477–6488).

### 3.4 Costs per dose and per fully immunized child

The estimated total cost per dose, including vaccine and delivery costs from all levels of the health system, was $4.32 (95% CI: 3.72–4.93). This value varied significantly across regions, with higher costs in Mtwara and Morogoro regions than in Mbeya and Simiyu regions (Supplementary Appendix, Fig. S1).

The delivery cost per dose was estimated to be $1.15 (1.06–1.70) (Table 3). The estimated delivery cost per dose delivered was lowest at rural facilities which include nomadic people in their catchment population ($1.00, 95% CI: 0.55–1.45), followed by rural facilities without nomadic people in their target populations ($1.19, 95% CI: 0.81–1.58) and finally urban facilities ($1.36, 95% CI: 0.42–2.31), though the differences across geographies were not statistically significant.

The estimated delivery cost per FIC was $24.28 (95% CI: 19.70–28.86). It was lowest in urban areas ($22.28, 95% CI: 19.70–28.86), followed by rural areas without nomadic populations ($22.97, 95% CI: 14.64–31.25), and with nomadic populations ($24.31, 95% CI: 18.90–29.72) (Table 4). As with the cost per dose, these differences were not statistically significant.

**Table 4:** Facility-level delivery cost per dose and FIC, by geography and delivery strategy (2016 US$).

	Delivery economic cost per dose (95% CI^1^)			Delivery economic cost per FIC^2^ (95% CI^1^)
	All delivery strategies	Facility-based delivery	Outreach-based delivery^3^	All delivery strategies
All health facilities (n = 51)	1.15 (0.83–1.47)	1.02 (0.72–1.32)	3.08 (1.41–4.75)	24.28 (19.70–28.86)
Urban areas (n = 17)	1.36 (0.42–2.31)	1.38 (0.38–2.38)	0.97 (0.31–1.64)	22.97 (14.64–31.29)
Rural areas without nomadic populations (n = 20)	1.19 (0.81–1.58)	1.00 (0.73–1.28)	3.16 (0.51–5.81)	24.31 (18.90–29.72)
Rural areas with nomadic populations (n = 14)	1.00 (0.55–1.45)	0.84 (0.53–1.15)	3.61 (2.70–4.52)	25.33 (17.75–32.90)

Notes: Table shows the average delivery cost per dose and per fully immunized child, by geographic area type (urban, rural without nomadic population, or rural with nomadic populations) and delivery strategy (facility-based or outreach-based delivery). Delivery costs include all costs apart from vaccines and vaccine injection supplies. The denominator includes all of the vaccine doses delivered to children under the age of 18 months, including: oral polio vaccine, Bacillus-Calmette-Guérin vaccine, Diptheria-Tetanus-Pertussis-Hepatitis B-Haemophilus influenzae type b vaccine, pneumococcal vaccine, rotavirus vaccine, and measles-rubella vaccine.

1 95% Confidence Interval.

2 Fully immunized child, defined as a child who received a second dose of the measles-rubella vaccine.

3 Outreach-based delivery was not used in all areas. In the 17 urban areas, 6 used outreach. In the 20 rural areas without nomadic populations, 13 used outreach. In the 14 rural areas with nomadic populations, 8 used outreach.

By delivery strategy, we found that outreach was more than three times more expensive than facility-based delivery on a delivery cost per dose basis ($3.08 (95% CI: 1.41–4.75) versus $1.02 (95% CI: 0.72–1.32)), and more expensive in rural areas than in urban areas (Table 4).

There were no mobile sessions conducted by any of the sampled facilities during the year of our study. If we assume all facilities involved in outreach would also conduct mobile delivery, this means 27 facilities cancelled planned mobile sessions, anecdotally understood to be due to challenges with funding for delivery costs at district level. Therefore, we do not report any results on the costs of mobile delivery.

**Fig. 1:**
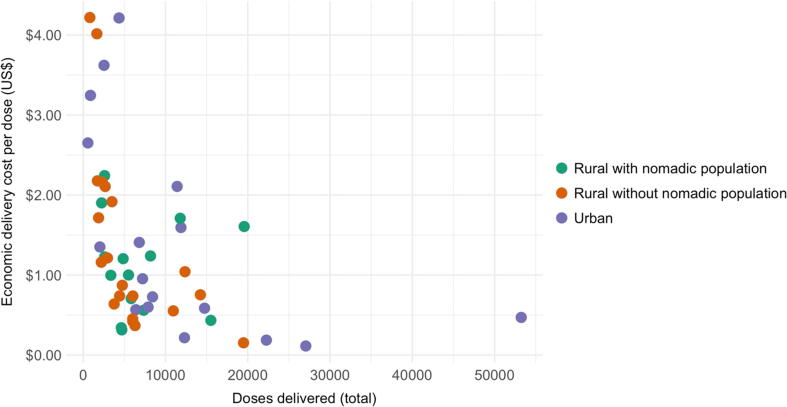
Facility-level delivery cost per dose (2016 US$) vs. number of doses delivered. Notes: Figure shows the association between facility-level delivery volume (for all vaccines given to children up to 18 months of age) and the facility-level delivery cost per dose. Each point represents one health facility. Colors indicate the geographic area of the facility.

Fig. 1 shows the negative association between facility-level delivery volumes and facility-level delivery cost per dose for the 51 facilities in the analytic sample. In the full sample, a 10% increase in delivery volume was associated with a 4.5% (95% CI: 3.4–5.5) decrease in the delivery cost per dose. The association was stronger among facilities in rural areas without nomadic populations (5.7%, 95% CI: 4.4–6.7) and urban areas (4.7%, 95% CI: 2.8–6.2) than in rural areas with nomadic populations (1.5%, 95% CI: −4.6 to 5.0).

### 3.5 Cost line items

**Fig. 2:**
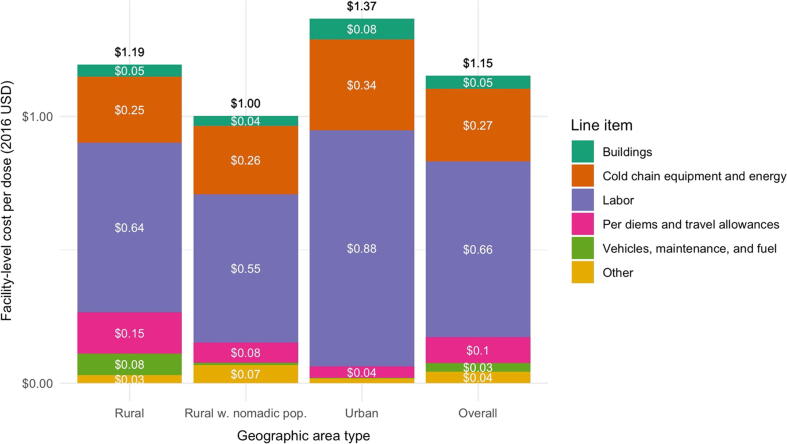
Facility-level delivery cost per dose, by line item. Notes: Figure shows the breakdown of the facility-level cost per dose by line item. The first three bars show the breakdown for facilities in a particular type of geographic area (rural without nomadic population, rural with nomadic population, and urban), and the final column shows the costs overall. For simplicity, chain equipment and energy costs are combined into one line item; vehicles, maintenance, and fuel are combined into one line item; and other supplies, other equipment, and other capital items are combined into one line item. The white text shows the average amount allocated to each line item, and the black text shows the total cost per dose incorporating all line items.

The main cost driving line items were labor, accounting for 57% of delivery costs per dose, followed by cold chain equipment and energy (23%), and then per diems and travel allowances (10%) (Fig. 2). The labor cost per dose was higher in urban facilities ($0.88) than in facilities in rural areas without nomadic populations ($0.64) or with nomadic populations ($0.55).

### 3.6 Evidence-to-policy translation

The findings were first disseminated in high-level meetings with Ministry of Health, Community Development, Gender, Elderly and Children (MOHCDGEC) management, then more broadly at the country’s annual Joint Appraisal meeting in November 2019, where progress and performance of Gavi support was discussed. A subsequent workshop with national and subnational stakeholders from the MOHCDGEC IVD and the Department of Policy and Planning focused on how to translate the findings for policy and planning. For the cost evidence to be usable, technical support was requested for the development of briefers and advocacy messages to use with non-finance stakeholders, tailored to policymakers and funders at different levels of the health system. Additionally, support from a finance technical advisor was specified as critical to help various levels of the health system monitor and use the cost evidence through the next planning cycle. This would include informing and updating plans based on supplemental analyses, such as how to optimize among vaccine delivery strategies, extrapolate to different geographic settings and integrate with other health services economic data. Discussions were underway as of February 2020 regarding how to fulfill these needs for policy translation support, but progress had not been made since dissemination to integrate the evidence into the planning cycle underway.

## 4 Discussion

We estimated the costs of the routine immunization program in Tanzania, where no immunization costing study had been conducted for five years. Findings from this study can inform planning and decision-making for the immunization program as Tanzania prepares to transition out of Gavi support. We found that the total cost of the routine immunization program was $138 million (95% CI: 133–144). The estimated cost per dose (for all levels of the health system) was $4.32 (95% CI: 3.72–4.93), of which $1.38 (95% CI: 1.06–1.70) was for delivery costs (exclusive of vaccines and injection supplies).

Our unit cost estimates are similar to estimates from other Sub-Saharan Africa countries. Four recent studies from Benin, Ghana, Uganda and Zambia reported an economic cost per dose delivered ranging from US$0.75 to U$S3.18, including injection supply and labor costs but excluding vaccine costs [34], [35], [36], [37], [38]. Our estimate of the delivery cost per dose of $1.38 was at the lower end of this range. If we included vaccine injection supplies, for increased comparability with the other studies, the estimated unit cost would be $1.83. Our estimate is close to the modelled estimate of $2.23 per dose (95% CI: $0.80–$5.07) for Tanzania in 2018, from a recent study that used available cost evidence to generate modelled estimates of unit costs for country-years where empirical evidence was not available [39].

Facility-level unit costs varied widely across facilities in the study sample, from $0.83 to $7.11 per dose. The variation in unit costs suggests that some facilities are more efficient at delivering vaccines than others. We estimated a negative association between service delivery volume and unit costs at the facility level, consistent with the findings of other studies [40], [41], [42]. However, this result should not be interpreted causally. Given the design of our study, we cannot conclude whether increasing facility-level service delivery volume would lead to a decrease in costs; the cost of increasing coverage will depend on the intervention used to do so [43].

We found a large difference between the delivery costs associated with different delivery strategies. Outreach delivery was approximately three times more expensive per dose as facility-based delivery ($3.08 (1.41–4.75) per dose compared with $1.02 (0.72–1.32) per dose on average). While facility-based delivery had similar costs across different geographic area types, outreach was more expensive in rural areas with nomadic populations ($3.61, 95% CI: 2.70–4.52) and rural areas without nomadic populations ($3.16, 95% CI: 0.51–5.81) than in urban areas ($0.97, 95% CI: 0.31–1.64). This is unsurprising, given that outreach requires additional resources such as transport for the vaccination team. However, this did not translate into a significant difference in costs between rural and urban areas overall, likely because outreach-based delivery made up only a small portion of delivery in the sampled facilities (approximately 5%) and was only implemented in 21 out of the 51 facilities in our sample. This finding contributes to a growing literature on the question of how costs vary by service delivery strategy [44], [45].

In addition to the minimal outreach services, none of the planned mobile delivery sessions took place in the sampled facilities during the year of our study. This could be reflective of challenges with funding for delivery costs at district level. Mobile delivery is likely to be more expensive than outreach or facility-based delivery due to the greater distances to be covered and overnight stay required; this may increase the cost per dose or per fully immunized child in rural areas with nomadic populations. Had budgeted amounts for mobile delivery and outreach-based delivered actually been released, then more delivery using these strategies might have happened and the measured costs might have been different. Political and other decisions therefore have an important impact on costs. Allocating sufficient funding for delivery costs might help ensure mobile and outreach-based delivery can happen as scheduled, although increasing facility-based delivery at existing sites, particularly those in rural areas, may be preferable in terms of cost implications for the immunization program. Coverage would need to be monitored to ensure nomadic people and other populations living in hard-to-reach areas continue to access services if mobile delivery is discontinued.

Our study has several strengths. First, we collected data from a nationally representative sample, allowing us to make inferences about overall costs in Tanzania. Second, we used an existing data collection tool, the EPIC tool, increasing the comparability of our study with other immunization costing studies. Third, we used calibration weights to estimate unit and total costs at the facility, district, and region-levels. Though not commonly used in the immunization costing literature to date, this statistical approach has been recommended for use in multi-site costing studies such as this one. This method improves the precision of estimates by incorporating auxiliary information. Finally, we worked in close partnership with the Ministry of Health in Tanzania, to ensure that the study design met the Ministry’s needs.

Our study also has several limitations. First, we relied on administrative data, which can be prone to error. There were a large number of facilities with missing data. We dropped three facilities from the sample and, for facilities with missing data on doses delivered or on the breakdown between delivery strategies, we made assumptions to fill in some data gaps, primarily in the number of doses delivered. If the dropped facilities had different cost patterns from the retained facilities, this could bias our results. The impact is likely to be small, given the small number of omitted facilities relative to the overall sample size. The concordance of our findings with global evidence helps us remain confident in our results despite this limitation. Second, the delivery volume data we collected from the national level was missing information on several antigens. We estimated the total doses delivered by making inferences from the dose reporting in the facility sample. Third, because our sample of sites in rural areas with nomadic populations was purposive, our estimates of costs at this type of site might be biased. Our use of a calibration approach helps to adjust for bias related to delivery site size, but does not correct for all forms of bias that might occur through purposive sampling. Fourth, while our cross-sectional study design allows us to describe variation in costs across facilities, it does not enable us to estimate the causal effects of different factors on costs. To estimate causal effects, future studies should be implemented using causal inference designs, such as randomized experiments or quasi-experimental studies.

Despite responding to Tanzania’s need for evidence about the costs of immunization delivery, which is crucial for planning and advocacy as the country prepares to transition away from external support and mobilize more domestic resources for routine immunization, additional work is required to support the translation of the findings from this study for use in policy decisions and for planning and budgeting. This effort may be particularly challenging in Tanzania’s decentralized health system, with various ministries and offices involved in financing and delivering immunization services. Our experience confirms findings from other costing initiatives such as the ProVac Initiative (2004 to present, four program costing studies) and EPIC (2012–2014, six country costing studies) that efforts are needed to go beyond the generation of cost evidence to increase the actual usability of that evidence for country planning and decision making. The ICAN project explicitly set out with the purpose of generating evidence to inform country decisions, planning and budgeting, which led to policy and program-relevant research with results directed to be used in these country processes. However, as the project has come to a conclusion, ongoing technical support will be required to successfully transform and translate the cost evidence to the formats required for policy decisions and management and planning activities.

Lessons learned on this evidence to policy translation effort that may be of use to other researchers hoping to generate cost evidence for country use include the need to engage a broad group of stakeholders for help in interpreting the results, tailor messaging to different audiences, and identify the action steps to facilitate ongoing use, which likely include a set of supplemental analyses after the costing study ends to transform the costing data for specific use cases. From a process standpoint, a champion or a multi-stakeholder group to support use of the evidence in-country, as well as a more practical, task-oriented partner who can follow through on next steps, may help ensure policy translation happens [46].

## 5 Conclusions

In this study, we estimated the costs of the routine immunization program in Tanzania for a 1-year period from 2015 to 2016. These findings can be used to inform planning and decision-making for routine immunization services, and efforts to improve the efficiency of immunization service delivery going forward.

## Funding

This work was supported by a grant from the 10.13039/100000865Bill & Melinda Gates Foundation, Seattle, WA [grant number OPP1139192].

## Author contributions

AO, FM, KT, CS and KV developed the study design and approach and designed the data collection tools. KT and FM collected the data. KT, FM, CS and KV participated in data cleaning. EC conducted the statistical analysis. EC, KV and AO were involved in the interpretation of results. AO led the policy translation efforts. KV and EC drafted the manuscript. All coauthors reviewed and approved the final manuscript.

## Declaration of Competing Interest

The authors declare that they have no known competing financial interests or personal relationships that could have appeared to influence the work reported in this paper.
