# Supplementary material for: Immunization costs, from evidence to policy: Findings from a nationally representative costing study and policy translation effort in Tanzania
Source: Vaccine. 2020 Nov 10;38(48):7659–67. doi: 10.1016/j.vaccine.2020.10.004 (PMC7604567; doi:10.1016/j.vaccine.2020.10.004)
Supplement: Supplementary data 1 [file mmc1.docx]

**Supplementary Appendix**

Table S1: Data sources

| **Line item** | **Data source(s)** |
| --- | --- |
| Paid labor | Interview |
| Per diem and travel allowances |  |
| Vaccines | Record review |
| Vaccine injection and safety supplies | Record review |
| Other supplies | Record review |
| Transport and fuel | Record review, interview |
| Vehicle maintenance | Record review, interview |
| Cold chain energy | Record review, interview |
| Printing | Record review, interview |
| Utilities and communication | Record review, interview |
| Other recurrent costs | Record review, interview |
| Cold chain equipment | Observation, interview |
| Vehicles | Record review, interview |
| Lab equipment | Record review, interview, observation |
| Other equipment | Record review, interview, observation |
| Buildings | Interview, observation |
| Other capital items | Record review, interview, observation |

Notes: Data sources were the same for each level at which data was collected (facility, district, region, and national level). Interviews were conducted with health workers. Record review included review of registers and inventories.

Table S2: Unit costs of equipment inputs in Tanzanian Shillings

| **Equipment** | **Specification** | **Unit Cost**  **(Tanzanian Shillings)*** | **Lifetime** |
| --- | --- | --- | --- |
| Refrigerator (Electric/gas) | TCW 3000 DC | 6,276,268 | 10 |
| Refrigerator (Electric) | RCW 50EG | 4,000,000 | 10 |
| Refrigerator (Solar) | RCW 50EG | 4,000,000 | 10 |
| Refrigerator (Gas) | RCW 50EG | 4,000,000 | 10 |
| Ice lined refrigerator | Vestfrost MK 404 | 2,181,344 | 10 |
| Cold Box | RCW 25 | 1,696,601 | 10 |
| Vaccine carrier | Blowking | 17,368 | 20 |
| Ice Packs | Blowking - 0.3L | 977 | 15 |
| Fridge tag | Fridge tag | 14,112 | 6 |
| Generator | Perking | 20,000,000 | 5 |
| Voltage regulator | Voltage regulator | 38,000 | 5 |
| Air conditioner | Air conditioner | 750,000 | 10 |
| Desk top computer | Dell | 1,300,000 | 4 |
| Laptop computer | Toshiba | 1,200,000 | 4 |
| Printer | H Laserjet 177 Printer | 850,000 | 3 |
| Landcruiser | Landcruiser hardtop | 87,000,000 | 10 |
| Honda | Small CC125 | 3,000,000 | 3 |
| Honda | CC250 | 4,500,000 | 3 |
| Syringes 5 mil | - | 110 | Per syringe |
| Syringes 0.5 mil | - | 362 | Per syringe |
| Syringes 0.05 mil | - | 362 | Per syringe |
| Bicycle | - | 130,000 | 3 |
| Incinerator | - | 14,000,000 | 20 |
| Modified pit | - | 70,000 | 5 |
| Pit | - | 35,000 | 3 |
| Kerosene | - | 2,015 | per liter |
| Gas | - | 50,000 | per bottle |
| Electricity | - | 357 | per unit, kw/hr |
| Petrol | - | 2232 | per liter |
| Diesel | - | 2056 | per liter |
| Megaphone | - | 35,000 | 10 |
| Tables | - | 150,000 | 10 |
| Chairs | - | 60,000 | 5 |
| Cabinet | - | 300,000 | 10 |
| Bench | - | 40,000 | 5 |

*Costs are reported in 2016 Tanzanian Shillings. Unit cost data are sourced from interviews, records, and internet searchers.

Table S3: Salary scales for cost analysis (salary levels available in 2016 Tanzanian Shillings upon request)

| **Salary Scale** | **Salary Scale** | **Salary Scale** |
| --- | --- | --- |
| MD 2 | NO 1 | NO 1 |
| MD 1 | SNO | SNO |
| SMD | PNO 2 | PNO 2 |
| PMD 2 | PNO 1 | PNO 1 |
| PMD 1 | ALT | ALT |
| AMO 2 | SALT | SALT |
| AMO 1 | PALT | PALT |
| SAMO | LT 2 | LT 2 |
| PAMO 2 | LT 1 | LT 1 |
| PAMO 1 | SLT | SLT |
| CO 2 | PLT 2 | PLT 2 |
| CO 1 | PLT 1 | PLT 1 |
| SCO | LS 2 | LS 2 |
| PCO 1 | LS 1 | LS 1 |
| PCO2 | SLS | SLS |
| ACO | PLS 2 | PLS 2 |
| SACO | PLS 1 | PLS 1 |
| PACO | H Ass | H Ass |
| EN 2 | SH Ass | SH Ass |
| EN 1 | PH Ass | PH Ass |
| SEN | Ass Env HO 2 | Ass Env HO 2 |
| PEN 2 | Ass Env HO 1 | Ass Env HO 1 |
| PEN 1 | S Ass Env HO | S Ass Env HO |
| RN 2 | P Ass Env HO 2 | P Ass Env HO 2 |
| RN 1 | P Ass Env HO 1 | P Ass Env HO 1 |
| SRN | HO 2 | HO 2 |
| PRN 2 | HO 1 | HO 1 |
| PRN 1 | SHO | SHO |
| NO 2 | PHO 2 | PHO 2 |

Figure S1: Facility-level cost per dose, by district


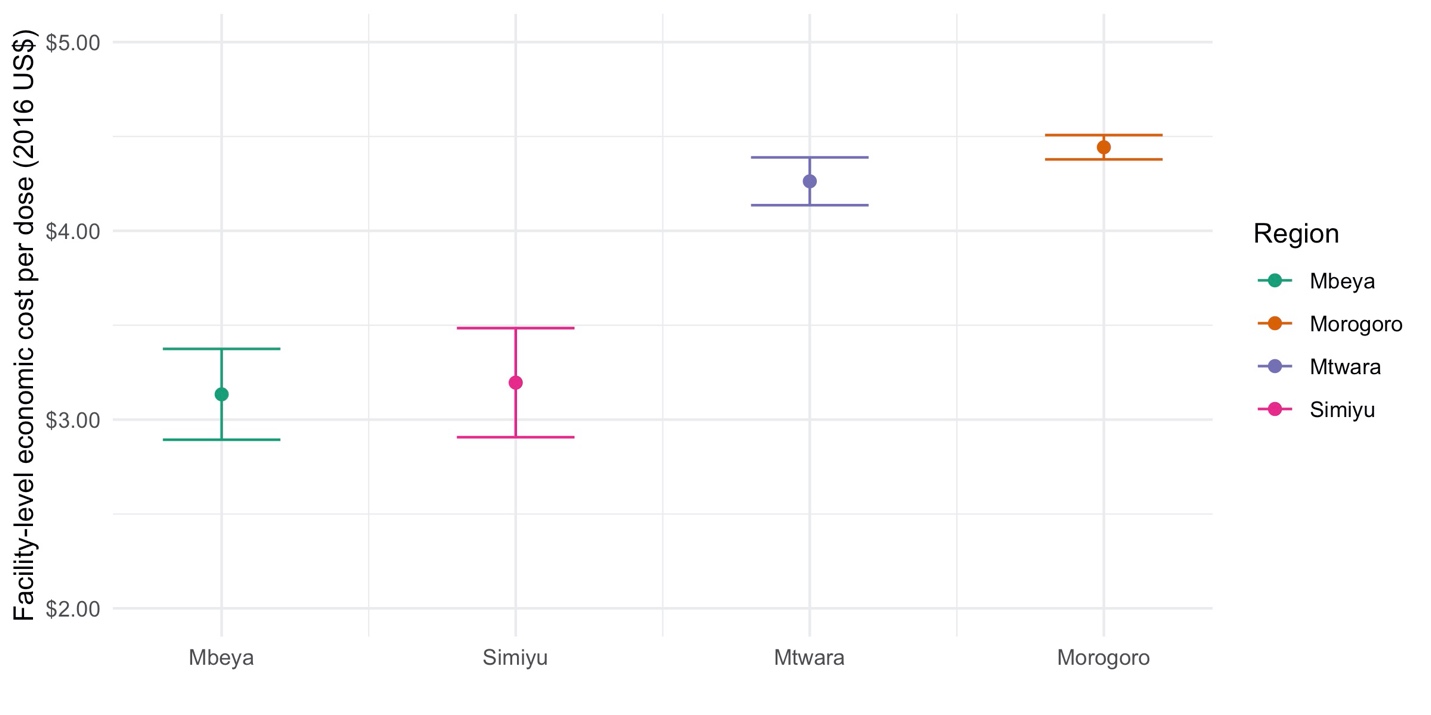


Notes: Figure shows the facility-level average cost per dose, by region. Cost per dose includes the costs of vaccines, vaccine supplies, and delivery costs. Error bars indicate 95% confidence intervals.
